# Editorial for the Special Issue “Cytochrome P450 (CYP) in Health and Disease”

**DOI:** 10.3390/biomedicines13040965

**Published:** 2025-04-15

**Authors:** Alevtina Grishanova

**Affiliations:** Institute of Molecular Biology and Biophysics, Federal Research Center of Fundamental and Translational Medicine, Timakova Str. 2/12, 630117 Novosibirsk, Russia; aiugrishanova@frcftm.ru

Cytochromes P450 (CYPs)—a large superfamily of unique heme-containing monooxygenases, are widespread among various organisms from all domains of life, including humans, animals, plants, fungi, and prokaryotes [1,2]. These enzymes participate in the oxidative metabolism of a wide range of lipophilic exogenous and endogenous substrates [3], in the biosynthesis of many physiologically important compounds [4], and in the biosynthesis of numerous secondary metabolites in plants, insects, and fungi [2]. These enzymes take part in the detoxification of many xenobiotics, such as pesticides, carcinogens, and environmental pollutants as well as prescription drugs [5]. Approximately 80% of reactions of CYP enzymes with clinically used drugs are carried out by a set of five CYP subtypes in the liver: CYP1A2, CYP2C9, CYP2C19, CYP2D6, and CYP3A4 [6]. The rate of metabolism by these enzymes is modulated by numerous endogenous and exogenous factors, including genetic polymorphisms, gender, age, ethnicity, the general state of health, and induction and inhibition by xenobiotics [4,7,8]. Additionally, CYPs are involved in several pathophysiological pathways, making these enzymes therapeutically relevant targets [9,10].

The Special Issue “Cytochrome P450 (CYP) in Health and Disease” addresses various aspects of CYP enzymes’ functioning that are related to the biochemistry of the enzymes, their inhibition and regulation, pharmacogenetics, and participation in various pathophysiological processes; these data are presented both in original research articles and in reviews.

The objective of one of the reviews in the Special Issue is to shed light on key roles of CYP3A enzymes, along with their unique characteristics in the metabolism of biologically active endogenous compounds and of numerous xenobiotics that are important in clinical pharmacology, as well as the involvement of these enzymes in diverse physiological and pathological phenomena. CYP3A enzymes are nonselective toward substrates and are unique in that they metabolize both endogenous compounds and a variety of xenobiotics, including prescription drugs. CYP3A enzymes are widely expressed in human organs and tissues, and the consequences of these enzymes’ activities play an important role both in the normal regulation of physiological levels of endogenous compounds and in various pathological conditions.

The scientific literature cited in this review reveals advances in the understanding of the integration of CYP3A enzymes in the vast and complex network of physiological processes of endobiotic and xenobiotic detoxification. The function of CYP3A enzymes is complex due to the activation of their genes by numerous endogenous and exogenous ligands and to a unique regulatory system involving CYP3A enzymes in many physiological and pathological processes within cells and tissues of the body. The review examines these aspects of CYP3A enzymes’ regulation under physiological conditions and their participation in the initiation and progression of various pathological conditions. First of all, diseases associated with the participation of CYP3A enzymes in the metabolism of bile acids, arachidonic acid, vitamin D, and sex steroids are reviewed. Various pathological conditions caused by inflammation and accompanied by changes in the expression and activity of CYP3A enzymes are analyzed, e.g., infections, inflammatory diseases, and cancer. Aberrant intratumoral expression of CYP3A enzymes is discussed too. Overall, the presented evidence suggests that the activation of *CYP3A* genes can be both beneficial and detrimental in diseases of various organs and tissues. The ultimate effects depend both on the context of a disease and on the nature of ligands of nuclear receptors that control the transcription of *CYP3A* genes.

To date, molecular mechanisms via which CYP3A enzymes take part in pathogenesis have been rigorously studied in only a few diseases. For example, the involvement of CYP3A5 has been demonstrated in carcinogenesis, in diseases associated with the participation of CYP3A enzymes in the metabolism of endogenous compounds, and in pathological conditions affecting the expression and activity of CYP3A enzymes.

Although much basic research has been conducted revealing the role of CYP3A enzymes in pathological processes, clinical studies are still insufficient, and further investigation is needed. The importance of such studies is explained by the fact that a consequence of changes in the activity of these enzymes is an alteration of the pharmacokinetics of prescription drugs.

The purpose of another review in the Special Issue is to formulate a clear notion about differential expressions of CYP enzymes in ovarian cancer, about the impact of this differential expression on the risk and prognosis of the disease, and about the influence of CYP enzymes’ polymorphisms on metabolism during chemotherapy and on the risk and prognosis of the disease. Here, data are examined on the expression of a large number of CYP enzymes during ovarian cancer that participate in the metabolism of xenobiotics, steroids, prescription drugs, and arachidonic and fatty acids, in the hydroxylation of retinoic acid, and in cholesterol biosynthesis. The authors analyze the significance of CYPs’ expression in terms of relations between CYP enzyme expression and immune infiltration and the patients’ survival.

Because individual expression of CYP enzymes in ovarian cancer may represent a potential therapeutic target, the feasibility of using metabolic expression of CYPs for the development of new selective treatments of ovarian cancer is discussed here. These treatments include the activation of prodrugs by means of CYP enzymes, immunotherapy targeting CYPs, and inhibitors of CYPs. Although there have been some interesting studies in this field, approaches to the design of prodrugs targeting selective expression of certain CYP enzymes in a tumor have not yet resulted in the approval of drugs for clinical use.

Another review deals with the structure and functional significance of the xenobiotic-metabolizing system in trematodes that cause helminth infections. Understanding the functionality of metabolic systems of these pathogens, including xenobiotic-metabolizing enzymes, is necessary due to considerable epidemiological significance of trematodes. In parasitic organisms, the need to adapt to conditions within a host in order to improve survival and self-reproduction is the driving force behind the regression of some organs and systems and high specialization of others. Vivid examples of biochemical adaptation to parasitism via simplification of the main metabolic systems are the simplification of cholesterol and fatty-acid biosynthesis pathways and the simplification of the xenobiotic metabolism system. For instance, in trematodes, the cytochrome P450 family is represented by a single gene.

In that paper, structural and functional organization of the xenobiotic metabolism-and-transport system in trematodes is described and compared with available data from other species.

Among various scenarios of functional and catalytic activity of P450 enzymes, the most likely, according to the authors of that review, is this enzyme’s evolutionary conservation, which may be related to the enzyme’s narrow specialization within a critically important endogenous process. This notion is supported by the fact that a comparison of differentially expressed genes among adult trematodes *Opisthorchis felineus*, *Opisthorchis viverrini*, and *Clonorchis sinensis* has not revealed significant differences in the set of genes encoding proteins of the detoxification system. This observation implies the conservatism of adaptation mechanisms of these helminths living inside a final host.

The review also touches on the importance of the xenobiotic metabolism system for the survival of helminths and on the potential usefulness of components of this system as targets for anthelmintic therapy. The topic of biochemistry of helminths’ P450 enzymes remains important and relevant and requires further investigation, because in this area, there are still unresolved issues regarding endogenous functions of the biotransformation system and regarding the influence of evolutionary simplification of the xenobiotic metabolism system on the functionality of this system.

The usefulness of one P450 cytochrome as a therapeutic target in another (fungal) infection is addressed in a research article about molecular cloning, purification, and evaluation of ligand-binding properties of *Candida krusei* cytochrome P450, which is an ergosterol-biosynthetic enzyme, i.e., steroid-14α-demethylase (CYP51). CYP51 is a key target of azoles: broad-spectrum pharmaceuticals used either as first-line drugs or as part of a combination therapy for the infectious disease (candidiasis) caused by yeasts of the genus *Candida*.

The main aims of that study were to determine whether the resistance of *C. krusei* to azoles is related to structural features of *C. krusei* CYP51—or is due to other factors—and to identify new chemical structures of *C. krusei* CYP51 active-site ligands that could be employed to develop a new generation of inhibitors.

A comparative analysis of structures between *C. krusei* CYP and CYP51 enzymes from other clinically important *Candida* species and a comparative analysis of their ligand-binding properties toward azole-containing antifungals were performed by those authors. They showed that the resistance of *C. krusei* to azoles is not due to structural features of CYP51 but to another mechanism. In an attempt to find new basal structures for the design of CYP51 inhibitors, the authors identified families of natural and synthetic steroid ligands—of the active site of *C. krusei* CYP51—that can be further investigated for the development of a new type of antifungal drug targeting CYP51.

If there is substantial variation in the ability to metabolize prescription drugs in a population, this situation may accelerate clinical consequences and affect the pharmacotherapy of patients. Such problems can range from the lack of efficacy to unexpected toxicity. Genetic polymorphisms of CYP enzymes may be responsible for interethnic and interindividual differences in the therapeutic efficacy of drugs. This Special Issue presents a study on genetic polymorphisms of CYP enzymes, which is an important step toward the understanding of genetic factors influencing a drug response in the Pashtun population of Pakistan: a country where pharmacogenomic research is insufficient.

The authors identified important pharmacogenetic variants in the *CYP2C9* gene that are associated with sulfonylurea-induced hypoglycemia. The CYP2C9*2 allele, known to correspond to low enzymatic activity, was found to be associated with sulfonylurea-induced hypoglycemia, whereas genotypes CYP2C9*1/*2 and CYP2C9*2/*2 proved to be more prevalent among patients with sulfonylurea-induced hypoglycemia compared with patients without this adverse reaction.

The importance of interactions between natural substances and cytochrome P450 enzymes has been elucidated by studies indicating that numerous compounds can act as inducers or inhibitors of P450 enzymatic activity. The aim of one of such studies presented in this Special Issue is to characterize the inhibitory effect of a sweet wormwood (*Artemisia annua* L.) extract on enzymes CYP2B6 and CYP3A4. *A. annua* has been used for nutritional and therapeutic purposes for many years. Its antimalarial, antimicrobial, cholesterol-lowering, antiviral, anti-inflammatory, antitumor, and anticonvulsant effects are known, but its impact on the activity of CYP enzymes has not been poorly studied.

The reason why sweet wormwood has a very wide spectrum of actions on various diseases is that it contains many biologically active families of compounds. The authors evaluated a methanolic extract of *A. annua* and demonstrated that it significantly inhibits enzymes CYP2B6 (almost by 90%) and CYP3A4 (by ~70%). At the same time, in various assays, a considerable decrease (by 46.8% and 38.2%) in the concentration of heme was noted; as the authors showed, this phenomenon is caused by binding of the extract’s reactive metabolites to heme, i.e., irreversible inhibition.

Knowledge about the irreversible inhibition of such important drug metabolism enzymes as CYP2B6 and CYP3A4 is important for clarifying potential clinical consequences of interactions of the *A. annua* extract with prescription drugs and food additives. Further studies can and should be aimed at characterizing the kinetics of the inhibition and at determining kinetic parameters for individual enzymes as well as the effect of *A. annua* extracts in vivo.

Determination of kinetic parameters of cytochrome P450–dependent reactions is crucial for the research on drug metabolism, for identification of inhibitors, and for assessment of drug–drug interactions. Electrochemical methods are top techniques in the enzymology of cytochrome P450 superfamily enzymes; the development of high-performance and convenient (in practical application) electrochemical assays for the identification and quantitation of metabolites from P450-dependent catalytic reactions is a topical urgent task of P450 enzyme enzymology and of experimental pharmacology. This Special Issue contains a study on the creation of a two-electrode system for assessing catalytic activity of cytochromes P450 using (i) electrodes with immobilized bactosomes and (ii) a voltametric analysis of a metabolite. As an example, the feasibility of this assay was demonstrated by means of hydroxylase activity of CYP2E1 toward chlorzoxazone. For electroenzymatic biotransformation of chlorzoxazone, the authors utilized a catalytic bioelectrode with bactosomes containing human CYP2E1, cytochrome P450 reductase, and human cytochrome b5 (CYP2E1BR) modified with a membrane-like compound (didodecyldimethylammonium bromide). A metabolite (6-hydroxychlorzoxazone) was detected by square-wave voltammetry based on direct electrochemical oxidation with the help of a measuring unmodified shielded electrode. Analyses of electrochemical properties of the bactosomes immobilized on electrodes, electrochemical assays of chlorzoxazone and of 6-hydroxychlorzoxazone, activity assessment and determination of steady-state kinetic parameters of the immobilized bactosomes, and an assay of the effect of applied reduction potential on electrocatalytic activity of the bactosomes were carried out next. All these results showed that the two-electrode approach to assessing the activity of bactosomes is practically convenient for identifying and quantifying metabolites formed during cytochrome P450–dependent catalytic reactions. An advantage of this approach is that it obviates multistage isolation of catalytic-reaction metabolites. Nonetheless, a possible limitation of the newly developed approach is that in experiments with CYP2E1 inhibitors, there is a risk of interference of oxidative potential of 6-hydroxychlorzoxazone with an inhibitor’s potential. The designed approach can be used with other CYP2E1 substrates, but in this case, the authors recommend investigating electrochemical properties of the substrates and possible products in advance.

Finally, the Special Issue also includes a bioinformatic study aimed at analyzing a gene coexpression network for identifying transcription factors (TFs) and long noncoding RNAs (lncRNAs) in the main organs featuring the expression of CYP3A enzymes—the human liver and small intestine—by means of a database called Genotype-Tissue Expression (GTEx) v8. Using (i) weighted gene coexpression network analysis (WGCNA), (ii) data about RNAs’ expression from GTEx v8, and (iii) multistep regression analysis, those authors built TF–lncRNA–CYP3A coexpression networks. Unlike methods that focus on individual genes or a few genes, WGCNA can transform gene expression data into coexpression modules, thereby providing insights into signaling and regulatory networks, and can identify RNAs having low levels but playing important regulatory roles in biological responses. Their analysis revealed reliable associations of several lncRNAs and TFs with the expression of CYP3A enzymes; these correlations differed between the liver and small intestine, indicating a contribution of lncRNAs to the different expression patterns of CYP3A enzymes and variable responses to drugs in each of the two organs. Inclusion of noncoding RNAs into the expression regulatory network of CYP3A enzymes revealed additional candidate TFs affecting the expression of CYP3A enzymes. Such research articles may serve as a guide for experimental studies on the regulation of CYP3A enzymes’ expression by lncRNAs and TFs in the liver and small intestine and ultimately can help to improve predictions of drug metabolism by CYP3A enzymes in the clinic.

Thus, the articles published in this Special Issue cover diverse research fields, show that a wide variety of aspects of cytochromes P450 are being studied, and should deepen our understanding of their role in health and disease. I hope that this group of articles will stimulate further basic and applied research in this area.
